# Elevated faecal ovotransferrin concentrations are indicative for intestinal barrier failure in broiler chickens

**DOI:** 10.1186/s13567-018-0548-4

**Published:** 2018-06-20

**Authors:** Evy Goossens, Griet Debyser, Chana Callens, Maarten De Gussem, Annelike Dedeurwaerder, Bart Devreese, Freddy Haesebrouck, Monika Flügel, Stefan Pelzer, Frank Thiemann, Richard Ducatelle, Filip Van Immerseel

**Affiliations:** 1 Department of Pathology, Bacteriology and Avian Diseases, Ghent University, Salisburylaan 133, 9820 Merelbeke, Belgium; 2 Laboratory of Microbiology, Department of Biochemistry and Microbiology, Ghent University, K.L. Ledeganckstraat 35, 9000 Ghent, Belgium; 3 Poulpharm BVBA, Prins Albertlaan 112, 8870 Izegem, Belgium; 4 Evonik Nutrition & Care GmbH, 33790 Halle, Westfalen Germany

## Abstract

Intestinal health is critically important for the welfare and performance of poultry. Enteric diseases that cause gut barrier failure result in high economic losses. Up till now there is no reliable faecal marker to measure gut barrier failure under field conditions. Therefore, the aim of the present study was to identify a faecal protein marker for diminished intestinal barrier function due to enteric diseases in broilers. To assess this, experimental necrotic enteritis and coccidiosis in broilers were used as models for gut barrier failure. Ovotransferrin was identified as a marker for gut barrier failure using a proteomics approach on samples from chickens with necrotic enteritis. These results were confirmed via ELISA on samples derived from both necrotic enteritis and coccidiosis trials, where faecal ovotransferrin levels were significantly correlated with the severity of gut barrier failure caused by either coccidiosis or necrotic enteritis. This indicates that faecal ovotransferrin quantification may represent a valuable tool to measure gut barrier failure caused by enteric pathogens.

## Introduction

Intestinal health is critically important for the welfare and performance of poultry. Enteric diseases that affect the structural integrity of the gastrointestinal tract lead to high economic losses due to reduced weight gain, poor feed conversion efficiency, increased mortality rates and greater medication costs [1, 2]. Coccidiosis and necrotic enteritis (NE) probably are the most common enteric diseases of poultry [3, 4]. In poultry, coccidiosis can be caused by multiple species belonging to the genus *Eimeria*, of which *Eimeria acervulina*, *E. maxima* and *E. tenella* are the most common species in intensively reared broilers. Lesions are found in the intestines at a specific place for each *Eimeria* species. In chickens, *E. acervulina* typically infects the duodenum, whereas *E. maxima* infects both the duodenum and jejunum and *E. tenella* infects the caecum [5, 6]. Depending on the species, the lesions can range from a limited malabsorptive enteritis (*E. acervuline*) to more severe inflammation of the intestinal wall (*E. maxima*) and even extensive caecal haemorrhage, leading to mortality (*E. tenella*) [7]. Furthermore, the presence of *Eimeria* species can also exacerbate the outcome of co-infection with bacterial pathogens such as *Clostridium perfringens* [8]. Indeed, the mucosal damage caused by these coccidial pathogens is an important predisposing factor for necrotic enteritis (NE) [3]. NE is the most common clostridial enteric disease in poultry, which typically occurs in broilers. The disease is caused by *C. perfringens* and can occur either in an acute clinical or as a mild subclinical form. Acute NE typically leads to a massive increase in flock mortality. The more common subclinical form is characterized by multifocal necrosis and inflammation of the small intestine with a significant decline in growth performance [1]. The reduction in performance is not only associated with impaired growth rate and feed conversion, but also with increased condemnation rates in broilers due to hepatitis at processing [9].

Both coccidiosis and necrotic enteritis can be present in a flock without clinical signs. Therefore, multiple birds have to be sacrificed for macroscopic examination of the intestine to diagnose disease. A faecal marker, or a set of markers, that can accurately detect intestinal inflammation and concomitant perturbation of the intestinal integrity at an early stage would be useful. Recently, there has been increased interest in research on intestinal permeability in chickens, resulting in different strategies to measure intestinal inflammation and concomitant gut barrier failure. However, none of the proposed measurements can be used as a marker for intestinal inflammation and barrier failure under field conditions as they are not easy to apply. The reasons are the requirement to use invasive techniques (e.g. oral administration of a marker that can be measured in the blood on a later timepoint [10, 11]) or the non-gut-specific nature of the markers (e.g. serum markers that can be elevated by non-gastrointestinal conditions as well [12–14]). Faecal markers may be more accurate in determining gastrointestinal inflammation and gut barrier failure [15]. The aim of this study was to identify a faecal protein marker for the detection of intestinal barrier damage and inflammation in broiler chickens. Therefore, proteome analysis was performed on faecal samples derived from animals under experimental necrotic enteritis challenge. The identified biomarker was validated using faecal and litter samples from birds with experimental necrotic enteritis or coccidiosis.

## Materials and methods

### Animal trials and sample collection

The experiments were approved by the ethical committee of the Faculty of Veterinary Medicine, Ghent University (NE trials; EC2013/180, EC2013/181 and EC2015/91) or the ethical committee of Poulpharm Bvba, Izegem, Belgium (coccidiosis trial; EC 201503). All animal experiments were carried out in accordance with the approved guidelines.

### Necrotic enteritis trials

An in vivo subclinical necrotic enteritis model based on previous studies was used [16]. Briefly, groups of 27 1-day-old Ross 308 broiler chickens were fed a diet rich in proteins and non-starch polysaccharides which predispose to the development of necrotic enteritis. A detailed diet composition was described elsewhere [16]. Other predisposing factors included the administration of Gumboro vaccine (either Nobilis Gumboro D78 (MSD Animal Health, Kempton Park, South Africa) or Poulvac Bursa Plus (Zoetis, New Jersey, USA), depending on the trial) to induce mild immunosuppression, and a tenfold dose of coccidiosis vaccine (either Paracox-8 (MSD Animal Health) or Hipracox (Hipra, Girona, Spain), depending on the trial) to induce predisposing intestinal damage. To induce necrotic lesions, animals were orally challenged with approximately 4 × 10^8^ CFU *C. perfringens* strain CP56 on 3 consecutive days, after which the animals were euthanized. At necropsy, lesion scoring in the small intestine (duodenum, jejunum and ileum) was performed as described by Gholamiandehkordi et al. [16] as follows: score 0 = no lesions, score 1 = congested intestinal mucosa, score 2 = focal necrosis or ulcerations (1–5 foci), score 3 = focal necrosis or ulcerations (6–15 foci), score 4 = focal necrosis or ulcerations (≥ 16 foci), score 5 = patches of necrosis of 2–3 cm long, score 6 = diffuse necrosis. Birds with a lesion score of 2 or more are classified as necrotic enteritis positive. In this study, samples were collected from three independent necrotic enteritis in vivo trials. From each trial, fresh distal colon content (further referred to as faecal samples) was collected from all birds and frozen at −70 °C. Samples from the first two trials were used for proteome analysis (see below). Samples from a third trial were used to validate the proteome results using ELISA (see below). From this third trial, also mixed litter was collected from each pen. After lesion scoring, the samples were grouped according to the disease severity of the animal, leading to the following disease severity groups: birds that received all predisposing factors but were not challenged with *C. perfringens*: negative control; birds challenged with *C. perfringens* but no necrosis: score 0 or challenged with *C. perfringens* and various severity degrees: score 2 (mild), score 3–4 (moderate) or score 5–6 (severe).

### Coccidiosis trial

Four hundred one-day-old Ross 308 broiler chickens were randomly allocated into groups of 20 birds and fed a standard broiler feed free from antibiotics and anticoccidials. To induce coccidiosis, 10 groups of chicks were inoculated orally with 15 × 10^4^ oocysts of *E. acervulina*, 4.6 × 10^4^ oocysts of *E. tenella*, 2 × 10^4^ oocysts of *E. maxima* and 1 × 10^4^ oocysts of *E. brunetti* at the age of 15 days. The birds in the other 10 groups remained uninfected and served as age-matched controls. Seven days after challenge, three chickens per group were euthanized for lesion scoring using the method of Johnson and Reid [17] and fresh distal colon content (further referred to as faecal samples) was collected. On the same day, one pool of fresh litter sample was collected from each group. All challenged birds showed macroscopically visible lesions of *Eimeria* infection, with a mean coccidiosis score of 5.11 ± 0.51, whereas only one out of 10 birds in the non-challenged control group was coccidiosis positive (coccidiosis score = 1). All samples were stored at −70 °C.

### Proteomic analysis of faecal samples from the necrotic enteritis in vivo trials

To identify protein markers that could be used as a biomarker for sub-clinical necrotic enteritis, five samples obtained from birds that were inoculated with *C. perfringens* and had a lesion score of 3–4 (moderate NE severity) were compared to five samples obtained from birds that received all predisposing factors but were not inoculated with *C. perfringens*.

### Sample preparation

Samples were prepared based on a previously described method for human stool samples, with some adaptations for chicken faeces [18]. Unprocessed faeces were thawed at room temperature. Approximately 0.5 g sample (wet content) was weighed and extraction buffer [50 mM Tris–HCl, 10 mM CaCl_2_, pH 7.8 and protease inhibitor cocktail (Roche Diagnostics Corporation, Indianapolis, USA)] was added till 10 mL. Samples were vortexed 10 times for 10 s, followed by a low-speed centrifugation step (10 min, 4 °C, 800 ×* g*) to remove large particles. The supernatant was transferred and the extraction was repeated twice.

Bacterial cells were lysed by sonication of the supernatants using a Barnson Digital Sonifier Model 250-D operating at an amplitude of 30% and a 1 s on, 1 s off cycle for 1 min. Subsequently the samples were cleared by high-speed centrifugation (10 min, 4 °C, 6000 × *g*) to remove cell debris.

After filtration (0.22 µm filter) the proteins were precipitated using 10% tri-chloro acetic acid (TCA) overnight at 4 °C. The precipitated proteins were collected by centrifugation (30 min, 4 °C, 16 000 ×* g*). The pellets were resuspended in 1 mL of extraction buffer, and transferred to a 10-kDa cut-off ultracentrifugation membrane filter tube (Amicon, Merck Millipore, Darmstadt, Germany). The proteins were washed with a minimum of 20 mL extraction buffer and concentrated until 0.5 mL.

## 1D gel electrophoresis and LC–MS/MS measurements

Protein concentration was measured by Bradford assay and 15 µg of protein solution was mixed with Laemlli buffer (62.5 mM Tris–HCl (pH 6.8), 10% glycerol, 1% SDS, 5% β-mercaptoethanol and 0.001% bromophenol blue). After boiling for 10 min, the reduced sample was loaded on a 1D SDS-PAGE gel. The molecular weight (MW) marker (Precision Plus Protein Standards, Bio-Rad, Nazareth Eke, Belgium) was loaded as reference to cut the lanes in five bands. The proteins were electromobilised at 150 V, the gel was then fixed and stained overnight with Coomassie brilliant blue G-250. After destaining in 30% methanol to obtain a clear background, the reduced and alkylated gel was sliced into five bands per lane. Each band was in-gel digested with trypsin and analysed via a fully automated LC MS/MS setup [19]. Briefly, 4 µL of a peptide solution was initially concentrated and desalted on a Zorbax 300SB-C18 trapping column (5 mm × 0.3 mm, Agilent, Santa Clara, CA, USA) at a 4 µL/min flow rate using a 2% (v/v) acetonitrile, 0.1% formic acid in water. After valve switching, the peptides were injected to and separated on a Zorbax 300SB-C18 analytical column, 150 mm × 75 µm (Agilent, Santa Clara, CA, USA), by a 30 min linear gradient ranging from 2% (v/v) to 50% (v/v) acetonitrile, 0.1% formic acid in water at a 300 nL/min flow rate. The eluting peptides were measured online on a LTQ-FT Ultra mass spectrometer (Thermo Fisher Scientific, Waltham, MA, USA). The FT-ICR mass analyser acquired MS scans at a resolution of 100 000 during the LC separation. The five most abundant molecular ions from each MS scan were automatically selected by the LTQ ion trap mass analyser. Subsequently, the precursor ions (charge state + 1 is rejected) were fragmented by collision-induced dissociation (CID), using normalised collision energy of 35.0%. After two occurrences, the precursor masses were excluded for 90 s.

### Protein identification

Peak detection from raw data was performed by ExtractMSn version 1.0.0.8 (Thermo, default parameters), which determines the monoisotopic m/z ratio value and charged status of the precursor ions along with the m/z values and the intensities of the fragment ions. Mascot Daemon (Matrix Science, London, UK; version 2.4.1) was used to search the retrieved peak list against the NCBI non-redundant protein database. Search parameters were assuming the digestion enzyme trypsin with a fragment ion mass tolerance of 0.3 Da, and a parent ion tolerance of 10 ppm. Oxidation of methionine and carbamidomethylation of cysteine were specified as variable modifications.

The resulting Mascot DAT-files were loaded in Scaffold (Proteome Software Inc., Portland, OR, version 4.4.1) and the MS/MS data were analysed using X! Tandem (The GPM, thegpm.org; version CYCLONE 2010.12.01.1). X! Tandem was set up to search a subset of the reverse concatenated database using the same parameters as Mascot, but glutamate and glutamine to PyroGlu and ammonia-loss of the N-terminus and oxidation of methionine and carbamidomethylation of cysteine were specified as variable modifications. Scaffold was used to validate MS/MS based peptide and protein identifications.

The peptide identifications were accepted if they could be established at higher than 99.0% probability by the Scaffold Local FDR algorithm. Protein identifications were accepted if they could be established at greater than 99.9% probability and contained at least two identified peptides. Protein probabilities were assigned by the ProteinProphet algorithm [20]. Proteins that contained similar peptides and could not be differentiated based on MS/MS analysis alone were grouped to satisfy the principles of parsimony. Proteins sharing significant peptide evidence were grouped into protein clusters. Quantification was based on exclusive spectral counting, a label free proteomics approach were the frequency of collecting a peptide precursor ion in MS/MS is used as a quantitative measure [21]. Spectral counts were normalized to the total number of spectra in the corresponding LC–MS run.

### Ovotransferrin detection by enzyme-linked immunosorbent assay (ELISA)

In order to confirm the ovotransferrin results obtained from the proteomic analysis of faecal samples from the necrotic enteritis in vivo trials, faecal samples from another, independent, necrotic enteritis in vivo trial were used. Eight samples from the non-challenged control birds (receiving all predisposing factors, but not challenged with *C. perfringens*) and eight samples per necrosis score group (either mild, moderate or severe NE) from challenged birds were selected. Also litter samples collected at the day of necropsy were included (one litter sample per pen, with in total three samples from pens with non-challenged birds and three samples from pens with challenged birds). Additionally, the ovotransferrin concentration was determined in both faecal samples and litter samples derived from the coccidiosis trial. Therefore, 5 litter samples from pens with non-challenged birds and 6 litter samples from pens with *Eimeria*-challenged birds were used. Additionally, 20 faecal samples from either *Eimeria*-challenged birds (*n* = 10, one sample from each group) or their non-challenged controls (*n *= 10, one sample from each group) were selected.

Unprocessed faeces or homogenized litter material were thawed at room temperature. The faeces or litter material (150 mg) was diluted in 1500 µL TBS (50 mM Tris, 150 mM NaCl, pH = 7.2) with protease inhibitor cocktail (P2714, Sigma-Aldrich). The samples were mixed by vortex (2 × 1 min). Proteins (supernatants) were collected after centrifugation (13 000 × *g*, 10 min, 4 °C) and were used in duplicate (1/50 dilution) in the ELISA (Chicken Ovotransferrin ELISA, KT-530, Kamiya Biomedical Company, Tukwila, USA). The ELISA was performed according to the instructions of the manufacturer.

### Ovotransferrin stability in faecal material

To assess the stability of ovotransferrin in chicken faeces, ovotransferrin (Conalbumin, Sigma-Aldrich) was spiked in two faecal samples from different birds to obtain a final concentration of 200 µg ovotransferrin per gram faeces. The spiked samples were aliquoted and incubated at room temperature for 0, 1, 6 or 24 h, proteins were extracted as described above and used in duplicate (1/50 dilution) on the chicken Ovotransferrin ELISA.

In order to elucidate whether the observed decline in ovotransferrin signal is due to proteolysis caused by proteases present in the faeces, a second spiking experiment was set up. Ovotransferrin was spiked in two faecal samples from different birds. However, before spiking a protease inhibitor cocktail (Sigma-Aldrich, P2714; inhibits serine, cysteine and metallo-proteases) was either or not added to the ovotransferrin. The samples were incubated for 0, 2 or 24 h at room temperature. Protein extraction and ELISA were performed as described above and the pH was measured after different incubation periods. The % recovery of ovotransferrin at each timepoint was calculated relative to the 0 h timepoint.

### Statistical analysis

Normality of the data was tested with the D’Agostino-Pearson normality test. For the proteomic analysis of samples from the NE in vivo trials, a Fisher’s exact test was performed on the total spectral counts of the samples from non-challenged birds or the samples from birds with lesion score 3–4. Hochberg–Benjamini correction was performed to correct for multiple testing. Differences in ovotransferrin levels between necrotic enteritis severity groups (as measured by ELISA) were calculated using an a Kruskal–Wallis test, followed by Dunn’s post hoc test. Differences in ovotransferrin levels between the *Eimeria*-challenged and the non-challenged control group were calculated using a Mann–Whitney test. The Spearman rank correlation was used to assess the relationship between the ovotransferrin concentration in the faecal samples and either the necrotic enteritis lesion score or the coccidiosis score. Results were reported as means and standard error of the means (SEM).

## Results

### Proteomic analysis and identification of differentially expressed proteins in necrotic enteritis samples

A total of 355,641 spectra were generated during the collection of MS/MS data from ten samples: five samples from birds that did not receive *C. perfringens* challenge (neg. ctrl) and five chickens with a lesion score 3–4. Using the NCBI non-redundant protein database 111,054 spectra (99.0% minimum, 0.01% decoy FDR) were identified. These peptides could be assigned to 1099 (99.9% minimum, 2 # peptides, 0.1% decoy FDR) proteins. Proteins sharing significant peptide evidence were grouped into protein clusters, and 707 protein clusters were detected.

Of these proteins, 155 were in common, 288 proteins unique for the samples from non-challenged birds and 500 proteins unique for the fraction with lesion score 3–4. 137 proteins were statistically more abundant in the samples from diseased birds. 60 proteins were present in 4 or more samples from birds suffering from necrotic enteritis.

Among the proteins that were more abundant in challenged birds was ovotransferrin. This protein was detected in all five samples from birds suffering from necrotic enteritis with an average spectrum count of 128. Additionally, ovotransferrin could be detected in 4 out of 5 samples from the non-challenged birds with an average spectrum count of 25. Therefore, there was an increased presence (*p *< 0.0001) of ovotransferrin in samples from birds suffering from necrotic enteritis.

### Faecal ovotransferrin concentration correlates with the severity of necrotic enteritis

**Figure 1:**
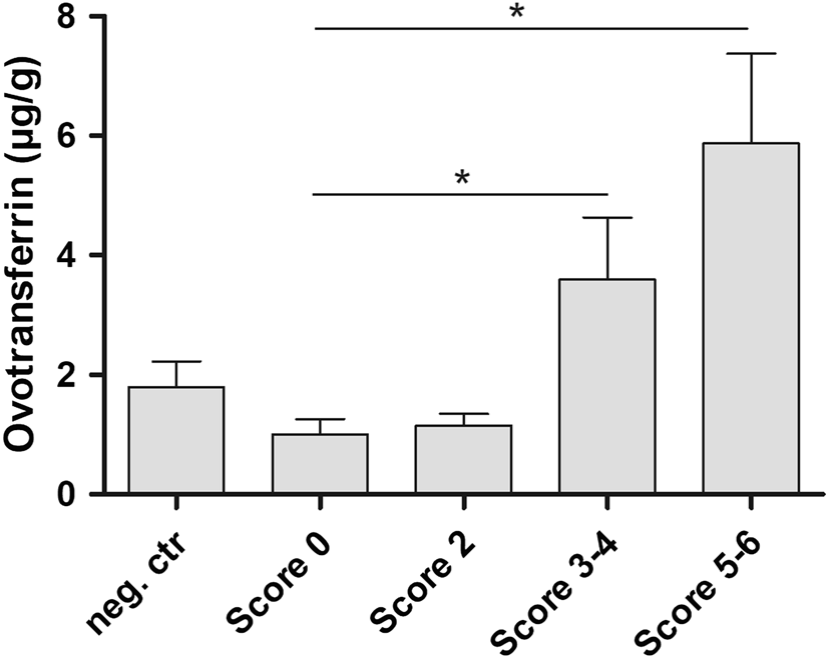
**Ovotransferrin concentrations in the faeces from birds derived from a necrotic enteritis in vivo trial.** The graph represents the ovotransferrin concentration measured by ELISA (mean ± standard error of the means) in faeces from birds that received all predisposing factors but were not challenged with *C. perfringens* (neg. ctr; *n *= 8) or from birds challenged with *C. perfringens* resulting in varying degrees of necrotic enteritis: no necrotic lesions (score 0; *n* = 8); mild intestinal necrosis (score 2; *n* = 8); moderate necrotic enteritis (score 3–4; *n *= 8) or severe necrosis (score 5–6; *n* = 8). *p < 0.05.

To confirm the results from the proteome analysis, an ovotransferrin ELISA was performed on samples from an independent NE in vivo trial. Most samples from birds suffering from either mild necrotic enteritis (score 2) or without intestinal lesions (both challenged and non-challenged control animals) showed a low signal. In samples from birds with more severe necrotic enteritis (necrosis score ≥ 3), significantly more ovotransferrin was detected than in samples from challenged birds that did not show intestinal disease (score 0) (Figure 1). Furthermore, there was a positive correlation between the necrotic enteritis disease severity and the ovotransferrin concentration in the faecal samples from the NE in vivo trial (*n* = 40, Spearman correlation coefficient = 0.53, *p* = 0.0004). This is not reflected in the litter samples from the NE trial, as no ovotransferrin could be detected in these samples.

### Ovotransferrin levels in the faeces correlate with the severity of coccidiosis

**Figure 2:**
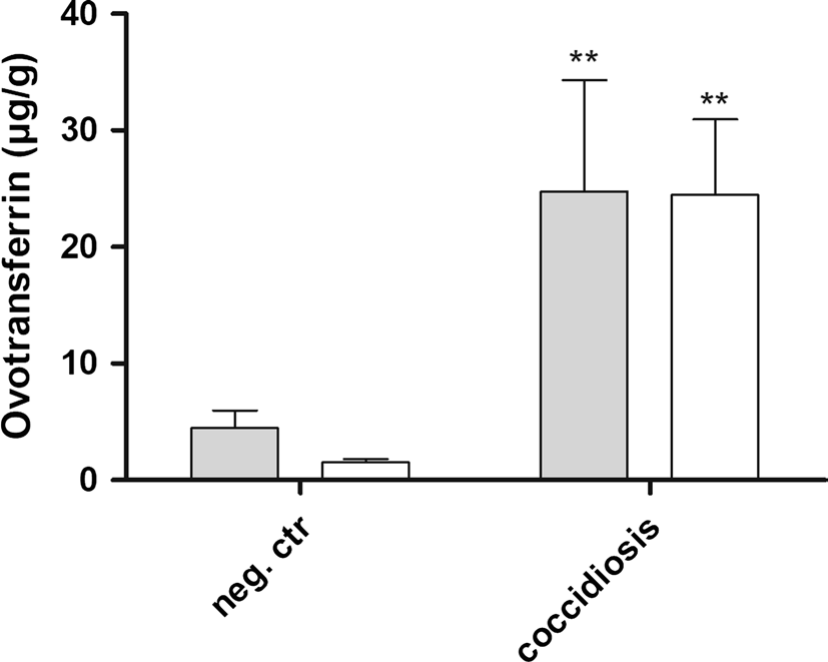
**Ovotransferrin concentrations in faecal and litter samples from experimental** ***Eimeria*-infected and control birds.** The graph represents the ovotransferrin concentration measured by ELISA (mean ± standard error of the means) in faeces (grey) or mixed litter (white) from experimental *Eimeria*-infected birds (coccidiosis; individual faeces samples: *n *= 10 or mixed litter samples: *n *= 6) or non-challenged control birds (neg. ctr; individual faeces: *n *= 10 or mixed litter samples: *n *= 5). Significant differences between the coccidiosis group and the non-challenged control group are indicated with **p < 0.01.

A challenge trial with different *Eimeria* species was used as a second model for gut barrier failure. The ovotransferrin levels in samples from *Eimeria*-challenged birds were elevated as compared to the non-challenged controls as measured by ELISA (*p* = 0.0029). Furthermore, there was a positive correlation between the coccidiosis score and the ovotransferrin concentration in the faeces (*n *= 20, Spearman correlation coefficient = 0.57, *p* = 0.0082). This difference in ovotransferrin levels was also reflected in the litter samples, where significantly higher ovotransferrin levels were detected in litter from *Eimeria*-challenged birds as compared to litter samples from non-challenged control groups (*p* = 0.0043) (Figure 2).

### Ovotransferrin is rapidly degraded by proteases in the faeces

**Figure 3:**
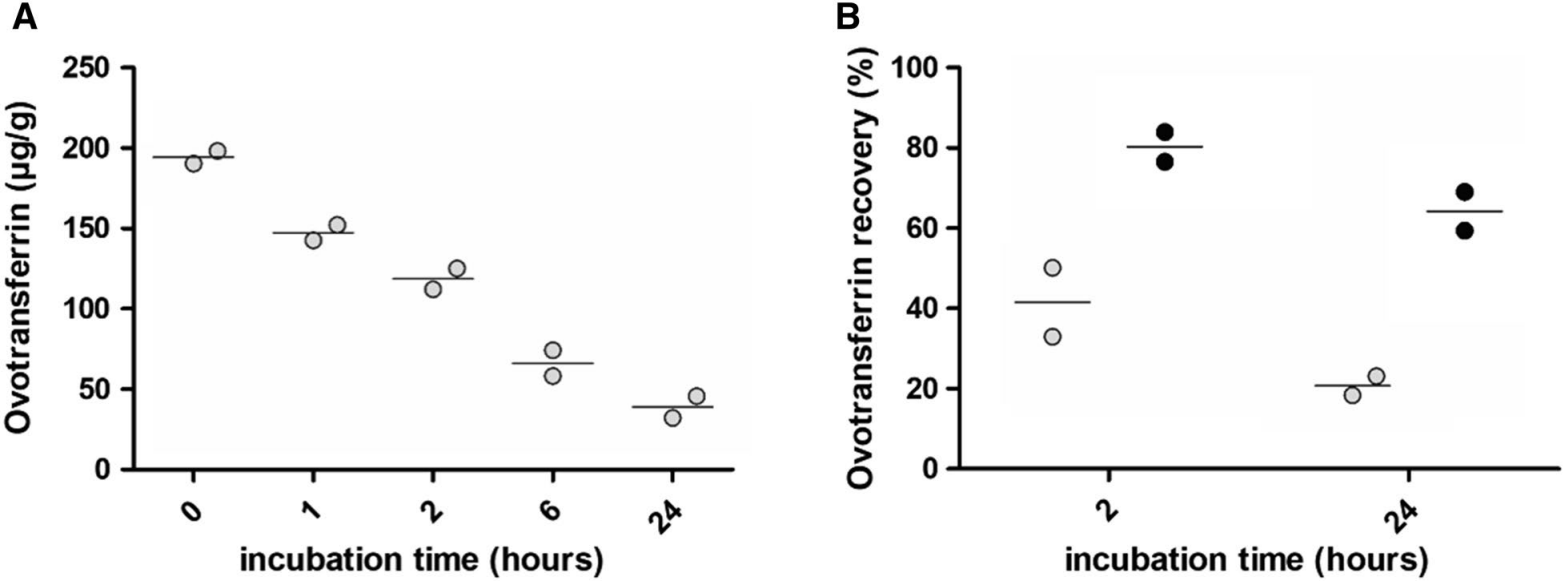
**Ovotransferrin stability in faeces from birds.** The ovotransferrin stability in the faeces was assessed by spiking two independent faeces pools from healthy birds with ovotransferrin at a final concentration of 200 µg/g. **A** The spiked samples were aliquoted and incubated at room temperature for 0, 1, 2, 6 or 24 h after which the ovotransferrin concentration was measured by ELISA. **B** The ovotransferrin stability in the faeces with (black) or without (grey) the addition of protease inhibitors was assessed after 0, 2 or 24 h incubation at room temperature. The % ovotransferrin recovery was calculated relative to the 0 h timepoint.

To address the stability of ovotransferrin in the faeces, ovotransferrin was spiked in two independent pools of faecal material originating from healthy broilers. The spiked samples were incubated at room temperature for different time intervals, leading to a gradual decrease of the ovotransferrin signal as measured by ELISA. The decline in ovotransferrin was similar for both pools of faecal material (Figure 3A). Neutralization of the proteases present in the faeces by addition of protease inhibitor cocktail increased the ovotransferrin stability in the faeces (Figure 3B). When no protease inhibitors were added to the pooled faecal content, only 51.29% of the ovotransferrin could be recovered after 2 h incubation, which decreased to a recovery of 20.36% after 24 h incubation. Addition of protease inhibitors increased the ovotransferrin recovery to 80.26% or 64.17% after 2 or 24 h incubation at room temperature, respectively.

## Discussion

A well-functioning and healthy gut is essential to ensure good broiler welfare and performance. Enteric diseases that affect the structural integrity of the gastrointestinal tract seriously affect bird performance, leading to economic losses. The availability of biomarkers for gut barrier failure would be of great value for the broiler industry (see Gilani et al. [10] and Ducatelle et al. [22] for a review). Faecal biomarkers for intestinal health are routinely used in human medicine as a preliminary screening tool and as an indicator of the severity of intestinal mucosal damage [15]. In birds, previous research mainly focussed on the detection of markers in blood and therefore no faecal markers were yet described to detect increased intestinal permeability caused by enteric diseases.

Ovotransferrin is an iron-binding glycoprotein that is synthesized both in the liver and the oviduct, which exhibits antimicrobial activity that can partially be attributed to its ability to bind and sequester iron (Fe^3+^), essential for bacterial growth [23]. Furthermore the 92-amino acid ovotransferrin peptide, OTAP-92, is capable of killing Gram-negative bacteria through membrane damaging mechanisms [24]. Ovotransferrin acts as an acute phase protein (APP) in birds [23, 25]. Acute phase proteins are blood proteins mainly produced in the liver, when triggered by different stimuli including trauma, infection, stress, neoplasia and inflammation (see Cray et al. for a review [26]). Changes in the circulating levels of APPs have been used as non-specific markers indicative of the health status in humans and animals. Indeed, serum ovotransferrin was previously described as a marker of infection and inflammation in chickens [14, 23, 25]. Moreover, ovotransferrin remains elevated as long as the inflammation persists, acting as immunomodulator and preventing microbial growth [23, 27, 28]. Chicken ovotransferrin can be classified as a moderate APP (one- to tenfold increase in circulating levels) [26]. Moderate APPs generally show prolonged increases, relatively slow declines and may be particularly associated with chronic inflammatory processes. Chen et al. [12] recently described a set of biomarkers to evaluate gut barrier failure in broiler chickens, which included the measurement of the APP α1-acid glycoprotein in serum. However, elevated circulating APP levels are likely not specific for gut barrier failure, as other, non-gastrointestinal diseases with inflammation can cause an increase in serum APPs as well [14, 15]. As an example, although elevated circulating ovotransferrin levels were previously measured in experimentally induced coccidiosis, this was also the case after pulmonary *E. coli* infection and vitiligo (an autoimmune-autoinflammatory disease), which are non-gastrointestinal diseases [25]. The measurement of markers in the faeces is likely more accurate in determining gut barrier failure.

In this study, faecal ovotransferrin was identified as a marker for gut barrier failure in broiler chickens. Indeed, elevated faecal ovotransferrin levels were measured in birds with either experimental coccidiosis or necrotic enteritis, which both cause intestinal gut barrier failure, using different approaches. First, proteome analysis of samples from different NE in vivo trials revealed that ovotransferrin was more abundant in samples from birds suffering from necrotic enteritis as compared to non-challenged birds. These results were confirmed via ELISA on samples from another, independent, NE in vivo trial. Additionally, elevated ovotransferrin concentrations were measured by ELISA in samples from *Eimeria*-infected birds as compared to their non-challenged controls. Faecal ovotransferrin levels were significantly correlated with the severity of gut barrier failure caused by either coccidiosis or necrotic enteritis, indicating that faecal ovotransferrin measurement might represent a valuable tool to measure gut barrier failure caused by enteric diseases. However, there was quite some variation in absolute ovotransferrin levels measured between the coccidiosis and NE in vivo experiments. In order to be used as a maker in the field, further research is needed to determine the ovotransferrin levels in the faeces from healthy as well as diseased birds under field conditions. Meanwhile, the measurement of ovotransferrin already represents an interesting tool to measure gut barrier failure under experimental settings, as it can assess efficacy of molecules or strategies that reduce intestinal disease.

Loss of plasma proteins into the gastro-intestinal tract is a well-known complication of a variety of enteric disorders which are associated with widespread loss of intestinal barrier integrity [29]. The proteome analysis revealed low levels of ovotransferrin in faeces of non-challenged birds. This is in accordance with a recent study of Marques et al. [30], showing low levels of ovotransferrin expression in epithelium and endothelium of the intestinal tract of healthy chickens. A wide range of infectious and non-infectious agents are known to damage the intestinal barrier in chickens, leading to gastrointestinal leakage and poor performance. Mild intestinal barrier damage will lead to reduced integrity of the epithelial layer, with consequent loss of fluid and ions towards the intestinal lumen [10]. More severe intestinal barrier damage will lead to translocation of micro-organisms and toxins from the intestinal lumen into the blood stream [31]. This translocation, together with the release of pro-inflammatory cytokines from the local inflammatory response in the gut, may trigger an acute phase response in the liver. The severe permeability defects in the gut epithelium may allow leakage of the APPs from the circulation into the gut lumen. This may explain why ovotransferrin is increased in the faeces only in case of widespread and severe intestinal barrier damage and was not elevated in samples from birds showing mild necrotic enteritis.

In order to be of value in the broiler industry, a marker should be detectable and predictive at flock level and not only at the level of the individual bird. Indeed, ovotransferrin could be detected in mixed litter samples from coccidiosis-challenged birds, whereas no ovotransferrin was measured in the litter from the control groups. However, no ovotransferrin was detected in the litter from the challenged birds in the necrotic enteritis trial. This might be attributed to the overall lower ovotransferrin levels measured in samples from the necrotic enteritis trial as compared to the coccidiosis trial, resulting in less ovotransferrin in the litter material. Additionally, ovotransferrin was rapidly degraded by proteases in the faeces, indicating that only relatively fresh material can be used for the assessment of gut barrier failure by measuring this marker. In order to circumvent this degradation problem, future research might focus on other, more stable APPs such as alpha-1 antitrypsin inhibitor protein, which is resistant to bacterial degradation or the effects of digestive enzymes within the gut lumen [32, 33].

In conclusion, the detection of ovotransferrin in the faeces represents an interesting strategy to measure gut barrier failure in poultry. Further research is needed to elucidate whether ovotransferrin release in faeces can be detected in other intestinal disease conditions in chickens and on the use of this faecal biomarker under field conditions.
